# Clinicopathological Characteristics and Survival Outcomes of Primary Signet Ring Cell Carcinoma in the Stomach: Retrospective Analysis of Single Center Database

**DOI:** 10.1371/journal.pone.0144420

**Published:** 2015-12-07

**Authors:** Xiaowen Liu, Hong Cai, Weiqi Sheng, Lin Yu, Ziwen Long, Yingqiang Shi, Yanong Wang

**Affiliations:** 1 Department of Gastric Cancer and Soft Tissue Sarcoma, Fudan University Shanghai Cancer Center, Shanghai 200032, China; 2 Department of Oncology, Shanghai Medical College, Fudan University, Shanghai 200032, China; 3 Department of Pathology, Fudan University Shanghai Cancer Center, Shanghai 200032, China; Duke Cancer Institute, UNITED STATES

## Abstract

**Purpose:**

To investigate the clinicopathological features and prognosis of signet ring cell carcinoma of the stomach (SRC).

**Methods:**

A total of 1464 gastric cancer patients who underwent curative gastrectomy from 2000 to 2008 at a single center were evaluated. Signet ring cell carcinoma (SRC) was defined as the presence of at least 50% signet ring cells in the pathologic specimen. The clinicopathological parameters and prognosis of SRC were analyzed by comparing with non-signet ring cell carcinoma (NSRC).

**Results:**

Of 1464 patients, 138 patients (9.4%) were classified as SRC. There were significant differences in gender, age, tumor location, TNM stage, p21 expression, and p53 expression between SRC and NSRC. The 5-year survival rates of SRC and NSRC were 36.2% and 49.5%, respectively. The prognosis of SRC was poorer than that of NSRC (*P* <0.001). Multivariate analysis showed that SRC histology was an independent factor for poor prognosis (*P* <0.001).

**Conclusion:**

Patients with SRC tend to present with a more advanced stage and poorer prognosis than patients with other types of gastric carcinoma.

## Introduction

Although the incidence of gastric cancer has been declining for several decades, it remains the fifth most common cancer and the third most common cause of cancer-related death worldwide [1,2]. Gastric cancer can be classified histologically into various types [3]. Signet ring cell carcinoma is a distinct histological type with cells containing abundant intracytoplasmic mucin [4]. It has been reported that 3.4% to 29% of gastric cancers are signet ring cell carcinomas [5–9]. Although some studies have reported on the clinicopathological features and prognosis of signet ring cell carcinoma of the stomach, results have been inconsistent, with some studies reporting a better prognosis compared with other gastric cancers [6,7,10], and others reporting a worse prognosis [9,11,12]. Therefore, the objective of this study was to investigate differences in clinicopathologic features and survival between signet ring cell carcinoma and other histological types of gastric cancer.

## Materials and Methods

### Patients

From 2000 to 2008, 1464 patients with histologically confirmed primary gastric adenocarcinoma underwent curative gastrectomy at the Department of Gastric Cancer and Soft Tissue Sarcoma, Fudan University Shanghai Cancer Center. Exclusion criteria for this study were as follows: (1) surgery status unknown; (2) vital status unknown; (3) incomplete pathological data. Signet ring cell carcinoma was defined as an adenocarcinoma with the presence of >50% of tumor cells (signet ring cells) with prominent intracytoplasmic mucins [13]. Data were retrieved from operative and pathological reports, and follow-up data were obtained by phone, outpatient and clinical databases. Written informed consent was obtained from all patients, and the study was approved by the Ethical Committee of Fudan University Shanghai Cancer Center.

### Preoperative evaluation and treatment

Preoperative examinations and staging was performed by endoscopic examination and computed tomography scan. Staging was carried out according to the American Joint Committee on Cancer (AJCC) TNM Staging Classification for Carcinoma of the Stomach (Seventh Edition, 2010). Gastrectomy was performed in accordance with the Japanese Classification of Gastric Carcinoma.

### Immunohistochemical staining

The expression of p21, p53, c-myc and EGFR in primary lesions were detected by immunohistochemistry. All primary antibodies and mouse monoclonal antibodies were purchased from Dako (Hamburg, Germany). The detailed sources, concentrations of antibody and positive sites were as follows: anti-p21 (clone SX118), 1:50 dilution, nucleus; anti-p53 (clone DO-7), 1:100 dilution, nucleus; anti-c-myc (clone 9E10), 1:100 dilution, cytoplasm; anti-EGFR (clone E30), 1:50 dilution, cytoplasm or membrane. The staining procedures followed supplier’ instructions. Negative controls were subjected to the same procedure except that the first antibody was replaced by PBS.

### Immunohistochemical Staining Scores

All slides were evaluated by two pathologists without knowledge of patients’ clinical data. The percentage of immunoreactive cells was graded on a scale of 0 to 4: no staining was scored as 0, 1–10% of cells stained scored as 1, 11–50% as 2, 51–80% as 3, and 81–100% as 4. Staining intensity was graded from 0 to 3: 0 was defined as negative, 1 as weak, 2 as moderate, and 3 as strong. The raw data were converted to an immunohistochemical score (IHS) by multiplying the quantity and intensity scores. An IHS score of 9–12 was categorized as strong immunoreactivity (+++), 5–8 as moderate (++), 1–4 as weak (+), and 0 as negative (-). On the final analysis, the cases with an HIS of less than 1 were classified as negative, and ≥ 1 as positive. These criteria were based on previously published reports [14].

### Follow-up

Follow-up of all patients was carried out according to our hospital’s standard protocol (every three months for at least 2 years, every six months for the next 3 years, and thereafter every 12 months for life) [14]. The check-up items included physical examination, tumor-marker examination, ultrasound, chest radiography, computed tomographic scan, and endoscopic examination. The median follow-up time was 64 months for living patients.

### Statistical analysis

The patients’ features and clinicopathological characteristics were analyzed using the χ^2^ test for categorical variables. Five-year survival rate was calculated by the Kaplan-Meier method, and differences between survival curves were calculated by the long-rank test. Independent prognostic factors were analyzed by multivariate survival analysis using the Cox proportional hazards model. The accepted level of significance was *P* <0.05. Statistical analyses and graphics were performed using the SPSS 13.0 statistical package (SPSS, Inc., Chicago, IL).

## Results

### Clinicopathological characteristics

Of 1464 patients, there were 1022 males and 442 females (2.3:1) with a mean age of 58 years. 138 patients (9.4%) had signet ring cell carcinoma and 1326 patients (90.6%) had non-signet ring cell carcinoma. By histological type, there were 35 well-differentiated tumors, 443 moderately-differentiated tumors, and 848 poorly-differentiated tumors in non-signet ring cell patients. By tumor location, 506 patients (34.6%) had tumors located in the upper third of the stomach, 248 (16.9%) had tumors in the middle third, 633 (43.2%) had tumors in the lower third, and 77 (5.3%) had tumors occupying two-thirds or more of the stomach. There were 111 (7.6%) patients with a family history of gastric cancer. The distribution of pathological stage was as follows: 346 (23.6%) stage I, 340 (23.2%) stage II, 778 (53.1%) stage III. Patients demographics were listed in Table 1.

**Table 1:** Patient Cohort.

	n = 1464	100%
Tumor subtype		
Signet-ring cell carcinoma	138	9.4
Other adenocarcinoma	1326	90.6
Sex		
Male	1022	69.8
Female	442	30.2
Age (yr)		
<60	767	52.4
≥60	697	47.6
Tumor size (cm)		
<5	902	61.6
≥5	562	38.4
Tumor location		
Upper third	506	34.6
Middle third	248	16.9
Lower third	633	43.2
Two-third or more	77	5.3
Venous tumor emboli		
Yes	524	35.8
No	940	64.2
Nervous invasion		
Yes	564	38.5
No	900	61.5
Serosa invasion		
Yes	676	46.2
No	788	53.8
Lymph node metastasis		
Yes	501	34.2
No	963	65.8
TNM stage		
Stage I	346	23.6
Stage II	340	23.2
Stage III	778	53.1
Family history of gastric cancer		
Yes	111	7.6
No	1353	92.4
P21 expression		
Positive	949	64.8
Negative	515	35.2
P53 expression		
Positive	1052	71.9
Negative	412	28.1
c-myc expression		
Positive	929	63.5
Negative	535	36.5
EGFR expression		
Positive	581	39.7
Negative	883	60.3

Clinicopathologic characteristics were compared between signet ring cell carcinoma (SRC) and non-signet ring cell carcinoma (NSRC). Signet ring cell carcinoma presented at a younger age (*P* = 0.014); presented more frequently in females (*P* = 0.003). Patients with signet ring cell carcinoma were more likely to present with stage III disease (*P* = 0.003) (Table 2).

**Table 2:** Comparison of the Clinicopathological Characteristics of Patients With Signet-Ring Cell Carcinoma (SRC) and non-signet ring cell carcinoma (NSRC).

Variables	SRC n = 138	NSRC n = 1326	*P*
Gender			0.003
Male	81	941	
Female	57	385	
Age (yr)			0.014
<60	86	681	
≥60	52	645	
Tumor size (cm)			0.851
<5	84	818	
≥5	54	508	
Tumor location			<0.001
Upper third	22	484	
Middle third	34	214	
Lower third	68	565	
Two-third or more	14	63	
Venous tumor emboli			0.501
Yes	53	471	
No	85	855	
Nervous invasion			0.602
Yes	56	508	
No	82	818	
Serosa invasion			0.344
Yes	69	607	
No	69	719	
Lymph node metastasis			0.325
Yes	96	867	
No	42	459	
TNM stage			<0.001
Stage I	35	311	
Stage II	13	327	
Stage III	90	688	
Family history of gastric cancer			0.876
Yes	10	101	
No	128	1225	
P21 expression			<0.001
Positive	66	883	
Negative	72	443	
P53 expression			0.009
Positive	86	966	
Negative	52	360	
c-myc expression			0.111
Positive	79	850	
Negative	59	476	
EGFR expression			0.292
Positive	49	532	
Negative	89	794	

### The expression of p21, p53, c-myc, and EGFR

**Fig 1:**
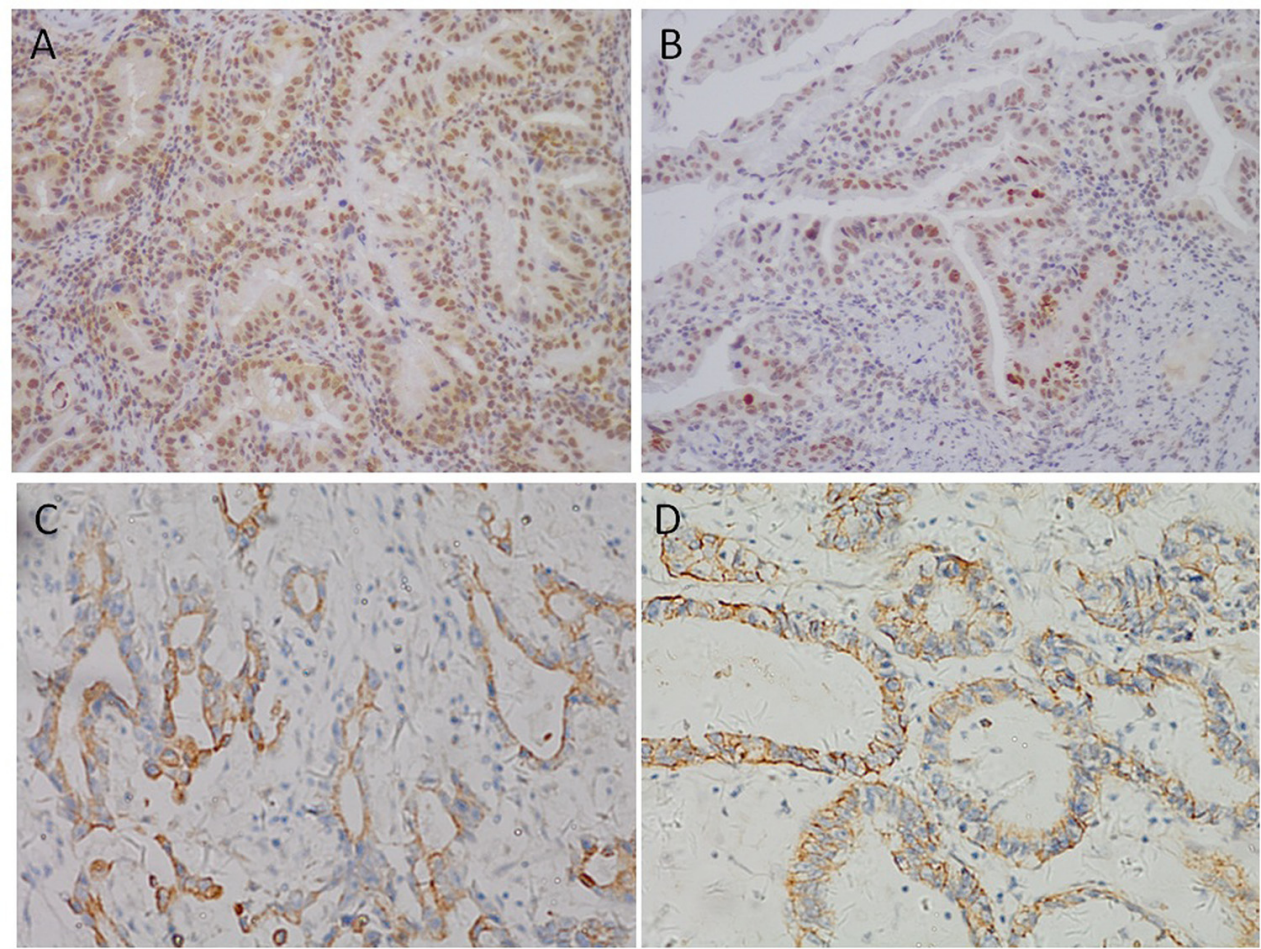
Positive expression of biological markers by immunohistochemistry in gastric cancer tissue. A) Positive expression of p21. B) Positive expression of p53. C) Positive expression of c-myc. D) Positive expression of EGFR.

The expression of p21, p53, c-myc, and EGFR were analyzed by immunohistochemical staining. Staining location was predominantly cell cytoplasm for c-myc, cell cytoplasm or membrane for EGFR, and nucleus for p21 and p53 (Fig 1). The positive expression rates of p21, p53, c-myc, and EGFR were 64.8%, 71.9%, 63.5%, and 39.7%, respectively. Relative expression of p21 and p53 was less in SRC than in NSRC, and the difference was significant.

### Univariate Analysis

**Fig 2:**
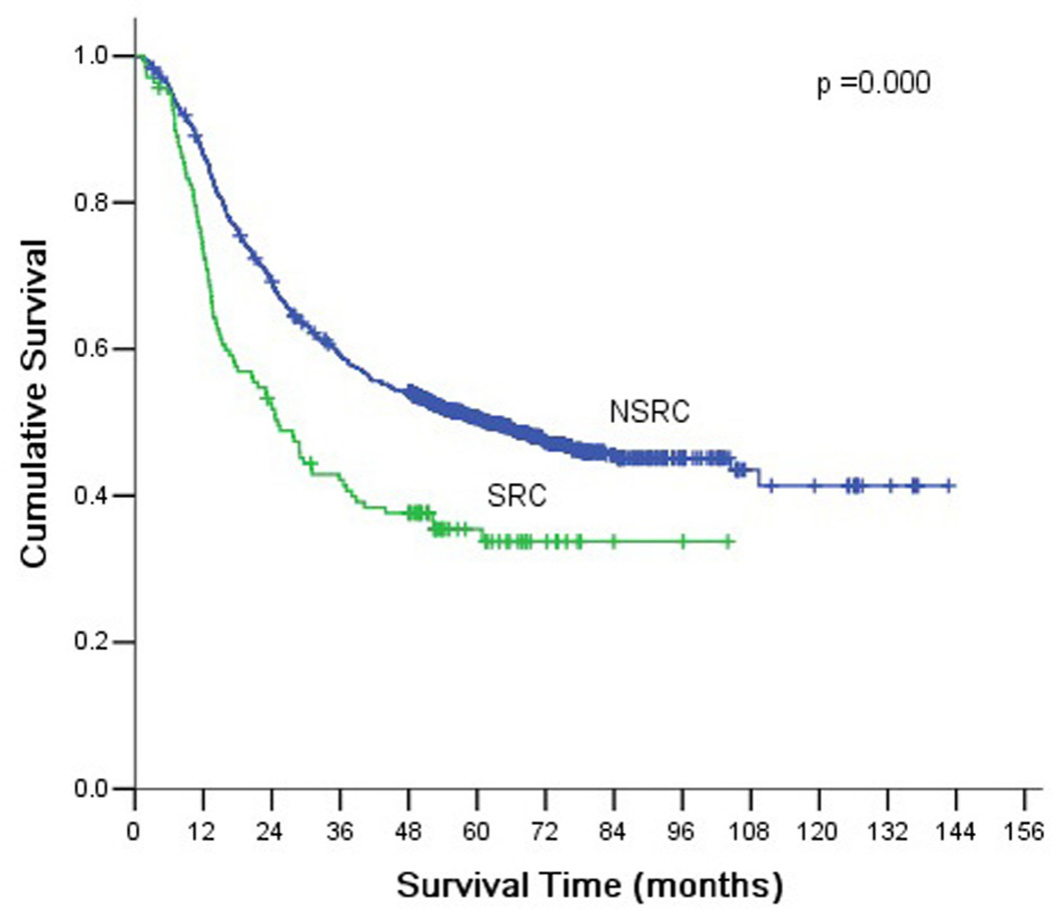
Kaplan-Meier survival curves by histological type. There were significant differences between SRC and NSRC (*P* <0.001).

The overall 5-year survival rate was 49% for all patients. The 5-year survival rates of SRC and NSRC were 36.2% and 49.5%, and the differences were statistically significant (Fig 2). In addition to tumor subtype, the significant prognostic factors were age, tumor size, tumor location, venous tumor emboli, nervous invasion, serosa invasion, lymph node metastasis, TNM stage, and EGFR expression (Table 3). In SRC, univariate analysis showed that age, tumor size, tumor location, venous tumor emboli, nervous invasion, serosa invasion, lymph node metastasis, TNM stage, and EGFR expression were significant prognostic factors (Table 4). In NSRC, univariate analysis showed that age, tumor size, tumor location, venous tumor emboli, nervous invasion, serosa invasion, lymph node metastasis, TNM stage, and EGFR expression significantly affected prognosis (Table 5).

**Table 3:** Univariate analysis of all patients by Kaplan-Meier method.

Variable	n	5-Year survival rate (%)	*P* value
Sex			0.989
Male	1022	48.0	
Female	442	48.6	
Age (yr)			<0.001
<60	767	53.5	
≥60	697	42.5	
Tumor subtype			<0.001
Signet ring cell carcinoma	138	36.2	
Non-signet ring cell carcinoma	1326	49.5	
Tumor size (cm)			<0.001
<5	902	57.5	
≥5	562	33.3	
Tumor location			<0.001
Upper third	506	38.1	
Middle third	248	44.4	
Lower third	633	61.3	
Two-third or more	77	19.5	
Venous tumor emboli			<0.001
Yes	524	26.7	
No	940	60.2	
Nervous invasion			<0.001
Yes	564	28.9	
No	900	60.3	
Serosa invasion			<0.001
Yes	676	28.4	
No	788	65.2	
Lymph node metastasis			
Yes	963	30.8	<0.001
No	501	81.6	
TNM stage			<0.001
Stage I	346	93.1	
Stage II	340	58.5	
Stage III	778	23.8	
P21 expression			0.497
Positive	949	47.1	
Negative	515	50.3	
P53 expression			0.901
Positive	1052	48.4	
Negative	412	47.8	
c-myc expression			0.391
Positive	929	47.6	
Negative	535	49.3	
EGFR expression			0.012
Positive	581	43.7	
Negative	883	51.2	

**Table 4:** Kaplan-Meier univariate analysis of patients with SRC.

Variable	n	5-Year survival rate (%)	*P* value
Sex			0.319
Male	81	34.6	
Female	57	38.6	
Age (yr)			0.012
<60	86	43.0	
≥60	52	25.0	
Tumor size (cm)			<0.001
<5	84	48.8	
≥5	54	16.7	
Tumor location			<0.001
Upper third	22	18.2	
Middle third	34	29.4	
Lower third	68	51.5	
Two-third or more	14	7.1	
Venous tumor emboli			<0.001
Yes	53	15.1	
No	85	49.4	
Nervous invasion			<0.001
Yes	56	21.4	
No	82	46.3	
Serosa invasion			<0.001
Yes	69	17.4	
No	69	55.1	
Lymph node metastasis			<0.001
Yes	96	16.7	
No	42	81.0	
TNM stage			<0.001
Stage I	35	91.4	
Stage II	13	53.8	
Stage III	90	12.2	
P21 expression			0.490
Positive	66	36.4	
Negative	72	36.1	
P53 expression			0.423
Positive	86	34.9	
Negative	52	38.5	
c-myc expression			0.202
Positive	79	31.6	
Negative	59	42.4	
EGFR expression			0.012
Positive	49	26.5	
Negative	89	41.6	

**Table 5:** Kaplan-Meier univariate analysis of patients with NSRC.

Variable	n	5-Year survival rate (%)	*P* value
Sex			0.960
Male	941	49.2	
Female	385	50.1	
Age (yr)			<0.001
<60	681	54.8	
≥60	645	43.9	
Tumor size (cm)			<0.001
<5	818	58.4	
≥5	508	35.0	
Tumor location			<0.001
Upper third	484	39	
Middle third	214	46.7	
Lower third	565	62.5	
Two-third or more	63	22.2	
Venous tumor emboli			<0.001
Yes	471	28.0	
No	855	61.3	
Nervous invasion			<0.001
Yes	508	29.7	
No	818	61.7	
Serosa invasion			<0.001
Yes	893	33.4	
No	433	82.7	
Lymph node metastasis			<0.001
Yes	867	32.4	
No	459	81.7	
TNM stage			<0.001
Stage I	311	93.2	
Stage II	327	58.7	
Stage III	688	25.3	
P21 expression			0.173
Positive	883	47.9	
Negative	443	52.6	
P53 expression			0.924
Positive	966	49.6	
Negative	360	49.2	
c-myc expression			0.510
Positive	850	49.1	
Negative	476	50.2	
EGFR expression			0.037
Positive	532	45.3	
Negative	794	52.3	

### Multivariate Analysis

Multivariate analysis was used to determine the independent prognostic factors for gastric cancer patients. Multivariate analysis by the Cox proportional hazard model found that tumor subtype, age, tumor size, venous tumor emboli, nervous invasion, TNM stage, and EGFR expression were independent prognostic factors for all the patients (Table 6). In SRC, multivariate analysis showed that age, TNM stage, and EGFR expression were independent prognostic factors. In NSRC, multivariate analysis showed that age, tumor size, venous tumor emboli, and TNM stage were independent prognostic factors for prognosis.

**Table 6:** Multivariate analysis of patients by Cox model.

Variable	*P* value	RR	95% CI
Age	0.000	1.310	1.132–1.516
Signet ring cell carcinoma	0.000	1.798	1.432–2.257
Tumor size	0.003	1.249	1.079–1.445
Tumor location	0.281	0.960	0.892–1.034
Venous tumor emboli	0.000	1.460	1.248–1.707
Nervous invasion	0.013	1.227	1.045–1.442
Serosa invasion	0.983	1.002	0.840–1.195
Lymph node metastasis	0.398	1.169	0.814–1.679
TNM stage	0.000	2.918	2.284–3.727
EGFR	0.038	1.168	1.009–1.352

### Comparison of Survival According to Stage Between SRC and NSRC Groups

**Fig 3:**
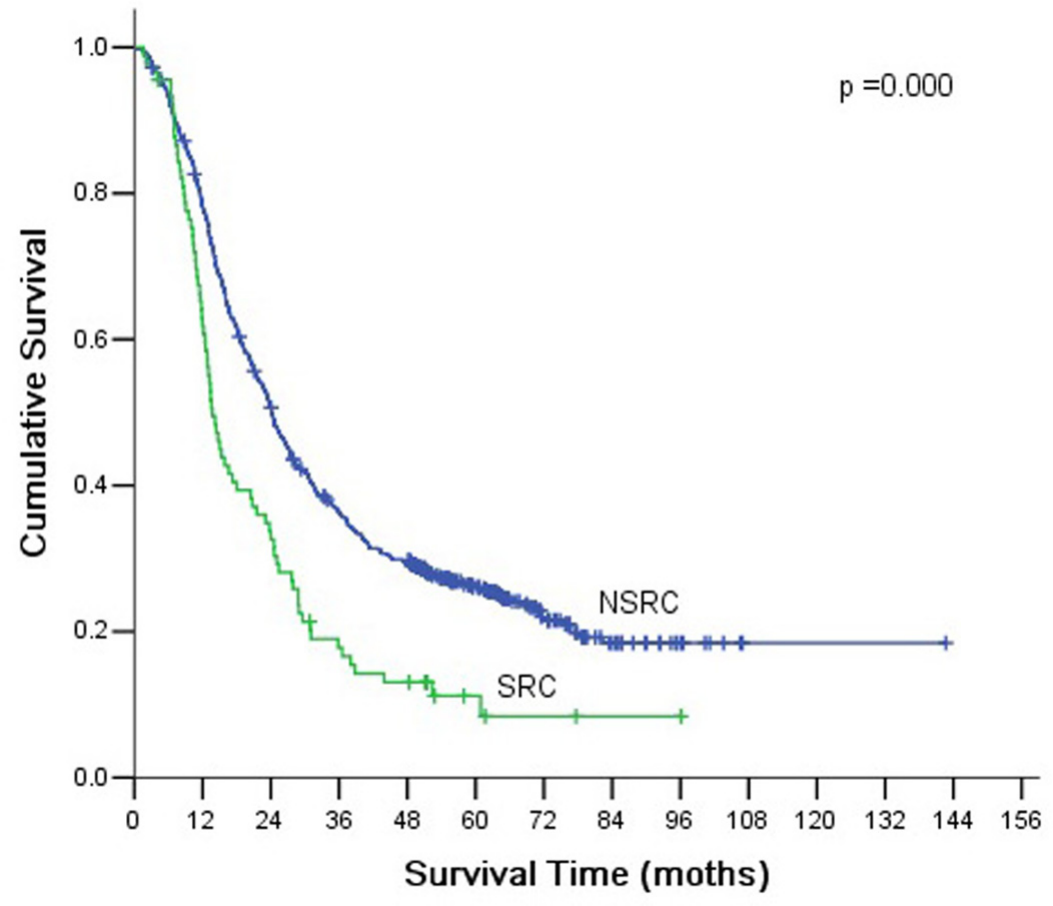
Comparison of survival according to tumor stage. There were significant differences between SRC and NSRC according to stage III (*P* <0.001).

According to the AJCC/TNM staging, gastric cancer patients are classified into stage I, II, or III. Histopathologically, tumors in each stage are classified as SRC or NSRC. There were significant differences of overall survival rates between SRC and NSRC in stage III (*P* <0.001, Fig 3).

## Discussion

The main findings of this study were as follows: (1) Signet ring cell carcinoma was an independent prognostic factor for five year gastric cancer. In particular, there was a significant difference in the survival of patients with stage III between SRC and NSRC. (2) There were differences in prognostic factors between SRC and NSRC, and EGFR expression was an independent predictor of poor prognosis for patients with SRC, but not for those with NSRC.

According to the Japanese gastric cancer classification system, tumors are classified by histological subtype as classical adenocarcinomas, signet ring cell carcinoma, mucinous adenocarcinoma, or other rare types [15]. Although some studies have shown that histological subtype is a key factor for tumor biology and prognosis, published results have been inconsistent [6, 7, 9–12]. In contrast to TNM staging, histological subtype is not incorporated in clinical classification systems.

In view of the inconsistent literature, we decided to evaluate the clinicopathological features and prognostic significance of SRC in our large single-center sample. In the current study, the incidence of SRC was 9.4% of gastric cancers, which was similar to incidence in previous reports [5–9]. We found that signet ring cell carcinoma had different clinicopathological features compared to other types of gastric carcinoma. SRC occurred more frequently in female and in younger patients than NSRC. This was also similar to the demographics reported in the previous studies [6, 8], though the exact reason remains unclear. It has been reported that histology may be influenced by sex hormones [16], but more research is needed to investigate the association between age, sex and gastric cancer histopathological type. Our sample also showed that SRC and NSRC tended to present at different anatomic locations, with SRC occurring more frequently in the middle third of the stomach. This was consistent with the findings of Ostuji et al. [7] and Zhang et al. [12]. Some previous studies have shown that SRC develops from the fundic glands, which are predominantly located in the fundus and body of the stomach [7, 17]. In contrast, Kim et al. [18] did not find differences in tumor location between SRC and NSRC. We also found that patients with signet ring cell carcinoma were more likely to present at more advanced stages, including a greater proportion of patients with stage III. Finally, on IHC biomarker analysis we found that the expression of p21 and p53 were significantly different between SRC and NSRC. In aggregate, the differences we identified between SRC and NSRC indicate that SRC may present a distinct and more aggressive disease.

In the current study, the 5-yr survival rate of patients with SRC was 36.2%, significantly shorter than patients with NSRC. Multivariate analysis showed that signet ring cell was an independent prognostic factors. However, this result could be related to the higher proportion of advanced stage tumors among SRC patients. In order to exclude the influence of disease stage at the time of presentation, we performed a subgroup analysis by tumor stage, which showed no significant differences in overall survival rates between SRC and NSRC in stage I and II. However, in stage III tumors, the prognosis was poorer in SRC than NSRC. These results were similar to previous studies [17, 19, 20]. Kim et al. [17] reported that the prognosis of early gastric carcinoma with SRC was similar to other types of gastric carcinoma. Li et al. [19] and Piessen et al. [20] found that the 5-yr survival rate of SRC was significantly poorer than that of NSRC in advanced gastric carcinoma.

In addition, we found that EGFR expression was an independent prognostic factor for patients with SRC by multivariate analysis, while it was not for those with NSRC. Given that SRC was not sensitive to common chemotherapeutic agents, the results of this study, indicating the association of EGFR expression and poor prognosis in SRC, may facilitate further development of agents targeting EGFR expression and clinical trials evaluating the role of those agents in SRC.

## Conclusion

SRC is a distinct type of gastric carcinoma in terms of clinicopathological features and prognosis. SRC presents with more advanced stage than NSRC, and carries a worse prognosis.
